# Safety and Efficacy Profile of *Echinacea purpurea* to Prevent Common Cold Episodes: A Randomized, Double-Blind, Placebo-Controlled Trial

**DOI:** 10.1155/2012/841315

**Published:** 2012-09-16

**Authors:** M. Jawad, R. Schoop, A. Suter, P. Klein, R. Eccles

**Affiliations:** ^1^Common Cold Centre and Healthcare, Cardiff University, Cardiff CF10 3XQ, UK; ^2^Cardiff School of Biosciences, Common Cold Centre, The Sir Martin Evans Building, Museum Avenue, Cardiff CF10 3AX, UK; ^3^D. S. H. Statistical Services GmbH, 85296 Rohrbach, Germany

## Abstract

*Objective*. To investigate the safety (risk) and efficacy (benefit) of *Echinacea purpurea* extract in the prevention of common cold episodes in a large population over a 4-month period. *Methods*. 755 healthy subjects were allocated to receive either an alcohol extract from freshly harvested *E. purpurea* (95% herba and 5% root) or placebo. Participants were required to record adverse events and to rate cold-related issues in a diary throughout the investigation period. Nasal secretions were sampled at acute colds and screened for viruses. *Results*. A total of 293 adverse events occurred with *Echinacea* and 306 with placebo treatment. Nine and 10% of participants experienced adverse events, which were at least possibly related to the study drug (adverse drug reactions). Thus, the safety of *Echinacea* was noninferior to placebo. *Echinacea* reduced the total number of cold episodes, cumulated episode days within the group, and pain-killer medicated episodes. *Echinacea* inhibited virally confirmed colds and especially prevented enveloped virus infections (*P* < 0.05). *Echinacea* showed maximal effects on recurrent infections, and preventive effects increased with therapy compliance and adherence to the protocol. *Conclusions*. Compliant prophylactic intake of *E. purpurea* over a 4-month period appeared to provide a positive risk to benefit ratio.

## 1. Introduction

The common cold is recognized as the most frequent disease in Western civilization and the number one cause of primary health care consultations [1, 2]. The costs of illness associated with noninfluenza infections are estimated at 40 billion USD, including direct and indirect costs. With the additional costs of illness caused by influenza, upper respiratory tract infections present a serious burden to humanity and to the economy [3, 4]. 

Colds comprise a syndrome of symptoms, typically with nasal complaints, cough, sore throat, and sometimes constitutional complaints, like headache, malaise, and fever [5]. The symptoms are typically self-limiting, and they represent a reaction to infection by Rhino-, Corona-, Adeno-, Respiratory Syncytial and (Para-) influenza virus [6]. 

The development of effective cold preventives is hampered by the multiplicity of viruses and the complex interplay between host and virus [7, 8]. For decades, intense research has focused on applications of broad-spectrum antivirals like interferons (*α*, *β*, or *γ*), capsid binding proteins, or soluble receptors directed against rhinoviral infection and/or replication. Some therapies showed efficacy in clinically induced infections but failed to significantly prevent colds in larger field studies that included multiple types of respiratory viruses. Nasal applications of interferons showed good preventative efficacy but were typically accompanied by adverse reactions like nasal bleeding [9]. 

Vaccination presents an effective method for managing seasonal influenza and respiratory, syncytial virus (RSV) in children. However, the efficacy of vaccination depends on the immunological fitness of the recipient and primarily in older individuals or those with chronic heart disease only insufficient immunity can be built up, resulting in a reduced immunity in this vulnerable population [10, 11]. 

Another method for preventing cold infections is to modulate the immune system [12]. In this context, *Echinacea* plays an important therapeutic role [13]. For several decades, *Echinacea* has been used to prevent colds and the flu [14]. Despite its worldwide acceptance, only limited data are available on its prophylactic efficacy. Long-term clinical trials that studied spontaneous colds, conducted by Schoeneberger, Schmidt and Schenk, Cohen et al., and Melchart et al., reported mixed results [15–18]. Three studies on artificially induced rhinovirus infections showed a trend toward preventing symptomatic cold episodes by *Echinacea* [19–21]. Generally, the prophylactic benefits reached significance when data were pooled in a meta-analysis, because single studies tended to have small sample sizes and undefined or low statistical power [22]. 

On the other hand, a good safety profile is mandatory for therapies that are designed to be taken over several months [23]. Considering the mild-to-moderate nature of the common cold, a potential preventive therapy by itself must induce only a minimal safety risk to produce a positive risk benefit ratio. In the predominant absence of side effects a sufficiently important difference of 20–32% is expected from cold treatments like vitamins and herbal extracts [24, 25].

The present study aimed to examine safety parameters of *E. purpurea* during long-term treatment. The study was designed to also investigate the efficacy profile with predefined, primary variables of efficacy and with an appropriate sample size based on power calculations. Overall, this study represented the largest clinical trial ever performed to test the safety and efficacy of *Echinacea* extract, and it was the first study to employ virus screening techniques.

## 2. Materials and Methods

### 2.1. Study Design

This study was a randomized, double-blind, parallel, placebo-controlled clinical trial conducted at the Common Cold Center in Cardiff University (United Kingdom). The study was conducted according to the declaration of Helsinki (2000), the international conference on harmonization, good clinical practice regulations, the association of the British Pharmaceutical Industry, and the human tissue authority. The trial received ethical approval from the local ethics committee by 28th July 2009 and, finally, from the medicines and healthcare products regulatory agency (MHRA) on the 2nd July 2009. The study was registered under the Eudra-CT number, 2009-012297-12. From October to November 2009, healthy participants were included in the study and were randomly allocated to receive either *E. purpurea* extract or placebo. At the inclusion visit, every participant received medication to cover 1 month of treatment and a diary for daily recordings of adverse reactions (“did you have any unusual or unexpected symptoms today?”), the presence and severity of cold-related symptoms and the use of any concurrent medication. Participants returned each month to the study center, and they returned any unused medication and the completed diaries. After checking compliance and the completion of the returned diary, a new treatment and diary were issued for another month. After the acceptance of an amendment to the study protocol, we also handed out three kits for self-collection of nasal secretions during acute cold episodes. Each kit contained a mid-turbinate nasal swab, suitable for self-collection, a vial that contained universal transport medium for storage at room temperature (COPAN, Brescia, Italy), and a bag for safe transport of the samples (DaklaPack, Oberhausen, Germany).

### 2.2. Treatment

The *Echinacea* product was the commercially available Echinaforce drops produced by A. Vogel Bioforce AG, Switzerland. Echinaforce was prepared by alcoholic (57.3% m/m) extraction from freshly harvested *E. purpurea* with a combination of 95% herba (DER = 1 : 12) and 5% roots (DER = 1 : 11). The sample was microbiologically tested and proven to be free of endotoxins. The batch used in this study (027643) was standardized to contain 5 mg/100 g of dodecatetraenoic acid isobutylamide, based on high-performance liquid chromatography measurements. Placebo drops were similar in shape, color, consistency, odor, flavor, and they contained the same amount of alcohol. The liquids were aliquoted into amber bottles and closed with a screw cap attached to an integrated, calibrated syringe for accurate dosing (0.9 mL/dose). Primary and secondary packaging was identical for the verum and placebo. 

### 2.3. Dosing

The therapy regimen was in accordance with the recommendation by the manufacturer. Participants swallowed 3 × 0.9 mL per day for illness prevention. This corresponded to 2400 mg of extract per day. During acute stages of colds, the participants were instructed to increase the dose to 5 × 0.9 mL per day; this totaled a daily dose of 4000 mg of extract. Each single dose was diluted in water and retained in the mouth for 10 s. This application method was expected to provide maximum local antiviral effects. Compliance was assessed at the monthly visits by weighing the returned bottles. Adherence to the recommended dosing was calculated based on the total prophylactic and acute dosing recorded in the diary. Overall, the method of administration reflected the traditional use of *E. purpurea*, and it provided accurate Echinaforce dosing.

### 2.4. Study Participants and Randomization

Participants were recruited via advertisements around the university campus. At first contact, respondents received an info-leaflet describing the trial. The study inclusion criteria were adults (≥18 years old) of good physical condition, that experienced ≥2 colds per year. The exclusion criteria were ineffective contraception; participation in another study; women that were pregnant or breast feeding; current cold infection; currently taking antimicrobial or antiviral medication; alcohol or drug abuse; psychiatric disorders; epilepsy; attempted suicide; planned surgical intervention; serious chronic disease that could influence absorption, metabolism, or elimination of the medication; known AIDS or other autoimmune diseases; diabetes type 1; corticosteroid-treated asthma; medicinally treated atopy or allergy; a known allergy to plants of the composite family (Asteraceae). Volunteers with clinically relevant laboratory abnormalities were dropped out after inclusion. All participants provided signed, informed consent.

A total of 755 subjects were included. Subjects were randomly allocated to receive treatment or placebo. The randomization code was prepared in block-sizes of 6 with the “RANCODE Professional 3.6” program. Each participant received treatment based on his/her identification number, which was allocated according to the time point of inclusion. Drugs were personally dispensed by the investigator or personnel authorized by the investigator. The randomization procedure was prepared by a statistician. The original randomization code was retained by the statistician in a sealed envelope, and one copy was conveyed to the investigator. Only in a case of emergency the investigator was permitted to open the envelope that contained the identification of a treatment.

The blinding of the study treatment was found to be adequate, when pretested in 79 test persons. In both treatment groups, nearly half of the participants believed that they were given the Echinaforce preparation (17 of 38 subjects (45%) with placebo and 19 of 41 subjects (46%) with verum). A total of 15 recipients of placebo (39%) and 17 (42%) with verum stated that they did not know which preparation they were given.

### 2.5. Sample Size Calculation

With at least 300 evaluable subjects in each group, assuming a 0.2 proportion of individuals with adverse drug reactions (ADRs) within each group, the upper limit of the observed one-sided 97.5% confidence interval of the difference between the placebo proportion, *π*P, and the Echinaforce proportion, *π*E, was expected to be less than 0.1 with 86% power. Assuming a 20% drop-out rate, where 20% of participants would not follow the study per protocol for the entire 4 months of treatment, we estimated that 750 subjects would be required for inclusion into the study. With 700 cumulated episode days in the Echinaforce group, an anticipated prophylactic effect of 25%, resulting in 875 episode days for the placebo group, the study had sufficient statistical power to show prophylactic benefits with *P* < 0.05.

### 2.6. Clinical Outcome Measurements and Statistical Analysis

Over the entire study period, all participants retained a diary to record AEs by answering the question “Did you have any unusual or unexpected symptoms today?” Moreover, at the monthly study visits, participants were interviewed about acute or experienced events by the study physician. The AE analysis included all AEs with a date/time of onset on or after the start date of the study treatment. The analysis excluded AEs with a date/time of onset that occurred before the start date or when information on the date/time of onset was missing. All AEs were coded with the lowest level terms from the latest installed version of the MedDRA Dictionary (V. 13.1). 

For AEs described by a physician(s), the lowest level term was chosen that best matched the physician's actual description. These lowest level terms were translated into preferred terms (PTs) and classified into a system organ class (SOC) employing the latest installed version of the MedDRA Dictionary (V. 13.1). Primary analysis was performed on the basis of the per protocol population.

At inclusion and exclusion visits, participants provided blood samples. These were processed to determine clinical chemistry, hematology parameters, and differential blood cell counts. Clinically relevant abnormalities that deviated from the normal range were flagged by the laboratory. The final safety criterion was the assessment, by participants and physicians, of therapy tolerability.

Causal relationship between recorded AEs and the study medication was rated by the physician as either “not related,” “unlikely,” “possible,” “probable/likely,” “certain,” “not assessable/unclassifiable,” “unknown,” or “not applicable.” AEs that were at least “possibly” related to the medication were considered adverse drug reactions (ADRs); these were included in the primary analysis of the per protocol collective (PP). With this respect, the proportion of patients with any ADRs was compared between groups to determine the non-inferiority of the treatment. To prove safety, there should be less than a 10% (non-inferiority limit) difference between the proportions of patients with ADRs in the Echinaforce and placebo groups. The alternative hypothesis (HA) of interest was to show non-inferiority by determining that the proportion of patients with ADRs in the Echinaforce group (*π*E) would be lower than the proportion of patients with ADRs in the placebo group (*π*P) plus delta (i.e., HA: *π*E < *π*P + *δ*). The alternative hypothesis was accepted when the upper 95% confidence limit (two-sided) of the difference in proportions between Echinaforce and placebo was lower than delta. For this study, delta (*δ*) was 0.1, corresponding to 10%. The occurrence of AEs was a secondary safety variable and was deduced from the safety collective.

The second question the participant answered in the diary, “Do you believe you have a cold today?” was answered yes or no. During acute colds, the symptoms “headache,” “chilliness,” “sneezing,” “nasal obstruction,” “nasal discharge,” “sore throat,” “cough,” and “malaise” were rated on a 4-point Likert scale with 0 or no entry = absence, 1 = mild, 2 = moderate, and 3 = severe symptoms. In addition, the participant indicated in the diary the daily intake of concomitant medication and/or therapy. This matrix was based on the work by Jackson and colleagues, who described the clinical features and symptoms of a virally induced common cold [1]. Their definition is currently accepted as the most valid method for differentiating a cold from isolated symptoms (like hay fever or allergies) that do not develop into the clinical picture of a cold. Thus, a cold episode was defined as a minimal total symptom score of 14 (summed over 6 consecutive days), and the participants believed they had a cold and/or reported rhinorrhea that lasted for ≥3 days. A set of three predefined prophylactic variables were analyzed in a confirmatory manner: (1) the total number of cold episodes, (2) cumulative episode days, and (3) comedicated cold episodes. The three parameters were analyzed individually with a chi-square test to determine whether the ratio of cumulated events (i.e., cold episodes) in the treatment groups corresponded to the ratio of the underlying group samples. The primary efficacy analysis focused on episodes with durations <9 days that occurred in the intention to treat (ITT) population. The null-hypothesis was rejected when the chi-square statistic was >3.84, resulting in a *P* value <0.05. Likewise, the incidence of recurrent infections in the whole group was compared to the underlying group samples with a chi-square statistic analysis. The primary analysis of the preventive efficacy was in agreement with earlier work by Schmidt and Schenk or Tiralongo [16, 26].

Nasal secretions were collected during acute stages of colds. Samples were inserted into a transport vial and stored at the study site at −70°C. At the end of the clinical trial, the samples were analyzed for the presence of respiratory viruses (Provincial Health Services Authorities, PHSA; BC Center for Disease Control, Vancouver Canada). Briefly, RNA was isolated from the nasal secretions using MagMax Express 96 Nucleic Acid Extractor (Applied BioSystems, Foster City CA) and screened with a Respiratory Virus Panel. The FAST Multiplex panel (Roche Diagnostics, Basel, Switzerland) could detect the following viruses (Virus Type/Subtype): Influenza A H1/H3, Influenza B, Respiratory Syncytial Virus, Coronavirus 229E/OC43/NL63/HKU1, Parainfluenza virus 1–4, human Metapneumovirus, Entero-rhinovirus, Adenovirus, and human Bocavirus. Frequency ratios of every virus and of membranous virus infections between treatment groups were compared to the underlying group sizes using a chi-square test. 

All statistical analyses were performed with the SAS system (Version 9.2) and Testimate 6.4 (IDV, Datenanalyse und Versuchsplanung, Gauting/München). 

## 3. Results

**Figure 1:**
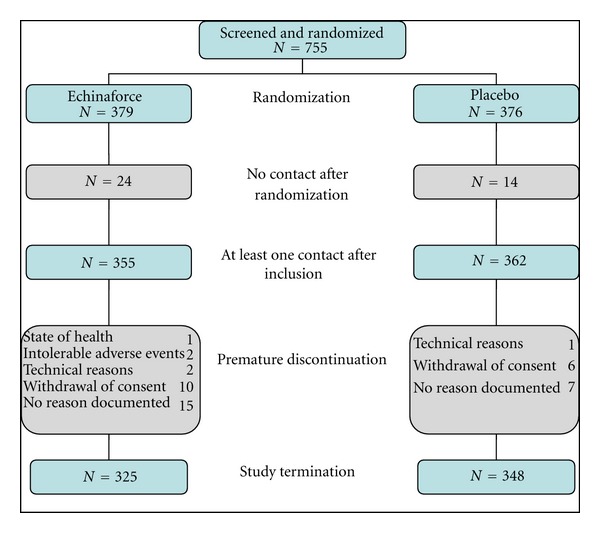
Flow diagram of participant disposition.

A total of 755 study subjects were screened and allocated into one of the treatment groups between October and November 2009. Of these, 673 subjects completed the study; the last patient visit was conducted in late April 2010. Eighty-two (10.9%) subjects discontinued the trial prematurely; of these, 38 were out of contact after randomization, 16 withdrew consent, 3 terminated the study due to technical reasons, 3 terminated due to intolerable AEs or deterioration of the participant's health, and 22 withdrew for no documented reason. A complete flow diagram of participant disposition is shown in Figure 1.

### 3.1. Demographic Data and Other Baseline Characteristics

The two groups were comparable with regard of age, gender, body weight, height, and body mass index (BMI). There was no noticeable difference between groups in anamnestic variables, including blood pressure or heart rate. The only variable that was significantly different between groups was the susceptibility to colds, measured as the number of colds experienced in the past. Participants in the placebo group were significantly less susceptible to infections than those in the *Echinacea* group (*P* < 0.05, Fisher's exact test) (see Table 1).

### 3.2. Analysis of Safety Variables

A total of 25 subjects in the Echinaforce group (9.0%) and 30 subjects in the placebo group (10.0%) experienced 27 and 30 ADRs, respectively. The percentage difference was −0.97%, with an upper limit of the one-sided 97.5% confidence interval of 3.6%, which is less than 10%. Consequently, Echinaforce was demonstrated to be noninferior to placebo in the incidence of ADRs as per protocol population.

A total of 293 AEs were reported by 177 subjects treated with Echinaforce and 306 AEs were reported by 172 subjects in the placebo group (safety collective). Four AEs in the Echinaforce group and 3 in the placebo group led to discontinuation of treatment (Table 2). No severe AE was observed with Echinaforce. One severe AE (glandular fever) occurred with placebo, and this required hospitalization.

Overall, no significant difference could be identified in the occurrence of AEs between groups, whether related or unrelated to the study drug (Fisher's exact test). This did not change when considering the total numbers, the system organ class, or the preferred terms (data not shown). 

In the hematological or biochemical measures no significant or clinically relevant changes from before to after Echinaforce treatment and in comparison to placebo were detected. No abnormalities were found after the 4-month exposure to Echinaforce. Previously reported safety concerns like induction of allergic reactions, leucopenia, or autoimmune diseases were not observed under *Echinacea* treatment [27].

About 64% of participants in the Echinaforce group and 71% in the placebo group assessed the tolerability of the medicine to be “good” or “very good.” There was no significant difference between groups.

### 3.3. Analysis of Prophylactic Efficacy

Efficacy was assessed concurrent with the safety variables during the long-term treatment with Echinaforce. A priori case definitions were made for sample size (calculation), statistical methodology, and measurements of probability or clinical end point.

The placebo group had a total of 188 cold episodes, with a collective duration of 850 episode days; in comparison, the Echinaforce group had 149 episodes with a collective duration of 672 episode days (ITT population). The difference of cumulated events (episodes and episode days) between the treatment groups each of 26% reached statistical significance for episode days (*P* < 0.05, chi-square test). A total of 65 recurring infections were observed in 28 participants with *Echinacea* and 100 episodes in 43 subjects under placebo treatment. The difference of 59% reached statistical significance as well (*P* < 0.05, chi-square test).

Concurrent medication was a significant factor in the present study. In the *Echinacea* and placebo groups, 58 and 88 episodes, respectively, were treated with aspirin, paracetamol, or ibuprofen. Thus, significantly more (+52%) cold episodes in the placebo group were additionally treated with pain medication (*P* < 0.05, chi-square test). The median of painkiller medicated cold episodes was 0 in the Echinaforce group and 1 in the placebo group.

A total of 201 nasal secretion samples were collected in the study; 86 in the *Echinacea* group and 115 in the placebo group, a difference with borderline significance (*P* = 0.0663, chi-square test). In 128 samples, the presence of a respiratory virus was confirmed. Throughout the whole study period, 54 viral infections were detected in the Echinaforce treated group and 74 were detected in the placebo group. Intriguingly, the strongest effect was seen with membranous viruses, like Corona-, Influenza-, Parainfluenza-, Respiratory Syncytial- and Metapneumovirus with 24 and 47 detected infections in the two groups (*P* < 0.05, chi-square test). In total, 14 recurring viral infections occurred under *Echinacea*, i.c. to 34 under placebo. 

In long-term studies (here, 4 months), compliance represents a sensible factor. Therefore, we specifically examined a population that took ≥100% of the recommended study medication for the entire study period. Eighty-eight compliant subjects in the *Echinacea* group reported 36 infections and 155 episode days; in comparison, in the placebo group, 58 episodes were reported with 268 episode days. This represented a 53% difference in the number of episode days. Despite the low denominators, this difference was highly statistically significant (*P* < 0.0001, chi-square test). Similar effects were observed in a group that was compliant in documentation (i.e., they reported at least one AE and/or one cold symptom in the diary during 4 months). However, these analyses were of explorative nature and served to substantiate the preventive effects of *Echinacea* in a compliant group. Moreover, the compliant group was less affected by confounders. 

## 4. Discussion

Prevention of mild-to-moderate diseases, like the common cold, requires therapies with satisfactory safety and efficacy profiles. The common cold is particularly in need of preventive treatments, due to its high frequency and high associated costs of illness [3]. It is assumed that the typical adult spends up to 2 years over a lifetime with cold symptoms [28]. Despite its prevalence and substantial research effort in the past, no specific preventive treatment has been developed to date that has a tolerable safety profile for use over the long term [7]. 

In the present study, safety and efficacy variables were analyzed over a collective total of 11,472 weeks or 2,868 months of prophylaxis from 717 subjects. We used a highly sensitive method to detect AEs, and we included the physician's experience to assess causality (ADRs). In addition, extensive laboratory tests were conducted to examine hematologic and metabolic parameters. 

The overall safety profile of Echinaforce was very good, based on the total AEs, the ADRs, and the laboratory measurements, within the treatment group and in comparison with placebo. In addition, the global tolerability assessments by the physicians and participants were quite positive. The fact that more than 75% mentioned that they would take the medicine again indicated that a 4-month treatment with Echinaforce was well accepted. Although the present data did not indicate any safety concern with *Echinacea* in a large population and over an extended period of time, we cannot fully exclude the possibility of rare and very rare adverse events with our data.

The study also assessed cold episodes using a highly accepted method developed by Jackson et al. [1]. The study was designed and large enough to show preventive efficacy with sufficient power. We employed a predefined and validated case definition, sample size calculations, statistical methodology, and measurements of probability according to a confirmatory approach [29]. Statistically significant differences between *Echinacea* and placebo were found for cumulative cold episode days and for comedicated episodes. Overall, we had expected about 1000 cold episodes, but we observed only 337 cold episodes. Although the treatment effect on the number of cold episodes was the same as for cumulated episode days, the statistical power was insufficient to detect a significant difference between groups for this parameter. 

A difference of 26% between groups in an open-field, long-term prevention study was comparable with a previous study on the effects of nasally administered interferons, and this difference can be considered clinically relevant [13, 24, 25]. In the present study, two covariates confounded the reported outcome significantly. First, participants in the *Echinacea* group had a higher susceptibility to colds than those in the placebo group. Second, participants in the *Echinacea* group reported less frequent use of classical pain medications and also of chlorphenamine, a codeine/cocodamol or pseudoephedrine. Adjusting for these covariates would most likely have resulted in an even higher effect, closer to the results reported from viral analyses and subgroups which shared similar anamnestic conditions. 

Previous studies have described the problems associated with assessing cold infections purely on subjective symptomatic grounds [7, 30]. Therefore, we aimed to substantiate our data with virus analyses in nasal secretions. Viruses were successfully detected via reverse transcriptase polymerase chain reaction (RT-PCR) in about 60% of samples. It was difficult to draw conclusions about specific viruses, due to the low overall number of samples. Therefore, we aimed to confirm our previous *in vitro* data, which showed that *Echinacea* had strong antiviral effects against membranous viruses [31, 32]. Indeed, upon pooling the incidences of Influenza, Parainfluenza, RSV, Metapneumovirus, or Coronaviruses, we found 47 total infections in the placebo group and only 24 infections in the *Echinacea* group (*P* = 0.0114, chi-square test). The individual reduction on infection depended of the respective virus type. These results were fully consistent with previous studies by Pleschka et al. that demonstrated similar antiviral effects with the same preparation used in the present study [31, 32]. 

## 5. Conclusion

The present work described the largest clinical trial to date that tested the safety and efficacy of *Echinacea* and investigated its risk/benefit in a long-term treatment. Prophylactic treatment with Echinaforce over 4 months appeared to be beneficial for many reasons. First, Echinaforce showed an advantageous safety profile; it did not induce any health risk above that reported with the placebo treatment. Second, prolonged treatment with Echinaforce was associated with significant therapeutic benefits.

Overall, the risk/benefit results from this clinical study suggested that long-term treatment with *E. purpurea* over 4 months can be recommended. 

## Figures and Tables

**Table 1:** Demographic and anamnestic data from participants in the safety collective at the inclusion visit.

Variables	Echinaforce (*N* = 355) means	Placebo (*N* = 362) means	*P* value
Age (years) (SD)	23.6 (7.8)	23.2 (7.2)	*P* > 0.05 (n.s)*
Body weight (kg) (SD)	67.7 (13.1)	69.5 (13.1)	*P* > 0.05 (n.s)*
Body height (cm) (SD)	167.5 (9.0)	168.1 (8.9)	*P* > 0.05 (n.s)*
Body mass index (SD)	24.1 (4.0)	24.5 (3.9)	*P* = 0.05 (n.s)*
Gender			
Female *N* (%)	244 (68.7)	227 (62.7)	*P* > 0.05 (n.s)**
Male *N* (%)	111 (31.3)	135 (37.3)	
Colds in the past; *N* (SD)	**3.0 (1.18)**	**2.8 (1.06)**	*P* < 0.05*

**Table 2:** Overview of adverse events (AEs) and adverse drug reactions (ADRs) that occurred during the study period in the safety collective.

	Echinaforce (*N* = 355)	Placebo (*N* = 362)	Total (*N* = 717)
Number (%) of participants with			
(i) adverse events	177 (49.9)	172 (47.5)	349 (48.7)
(ii) drug-related AEs^1^	35 (9.9)	35 (9.7)	70 (9.8)
(iii) serious AEs	0 (0.0)	1 (0.3)	1 (0.1)
(iv) AEs leading to treatment discontinuation	3 (0.8)	2 (0.6)	5 (0.7)
Number of events^2^			
(i) adverse events	293	306	599
(ii) drug-related AEs^1^	39	36	75
(iii) serious adverse events	0	1	1
(iv) AEs leading to treatment discontinuation	4	3	7

^1^AEs that were causally related to the study medication with ratings of certain, probable/likely, or possible.

^2^AEs were based on the Preferred Terms (PTs), each PT counted only once per participant.
